# Gold Nanoparticle-Enhanced Dual-Channel Fiber-Optic Plasmonic Resonance Sensor

**DOI:** 10.3390/s26020692

**Published:** 2026-01-20

**Authors:** Fengxiang Hua, Haopeng Shi, Qiumeng Chen, Wei Xu, Xiangfu Wang, Wei Li

**Affiliations:** 1 School of Integrated Circuit Science and Engineering, Industry-Education Integration College, Nanjing University of Posts and Telecommunications, Nanjing 210023, China; 1023223204@njupt.edu.cn (F.H.);; 2 School of Electronic and Optical Engineering, School of Flexible Electronics (Future Technology), Nanjing University of Posts and Telecommunications, Nanjing 210023, China

**Keywords:** sensor, surface plasmon resonance, localized surface plasmon resonance, photonic crystal fiber, gold nanoparticles

## Abstract

**Highlights:**

**What are the main findings?**

- A dual-channel D-shaped PCF-SPR sensor integrating a TiO_2_/Au bilayer with gold nanoparticles achieves strong hybrid plasmonic coupling and significantly enhanced field confinement.
- The optimized structure reaches a maximum wavelength sensitivity of 16,600 nm/RIU and improves sensing performance by 1.47× compared to conventional PCF-SPR designs.

**What are the implications of the main findings?**

- The demonstrated hybrid LSPR–SPP enhancement mechanism provides an effective strategy for boosting sensitivity in low-refractive-index biochemical environments.
- The proposed design offers a feasible pathway for developing next-generation high-performance optical fiber sensors for biochemical detection and environmental monitoring.

**Abstract:**

Surface plasmon resonance (SPR) sensors based on photonic crystal fibers (PCFs) hold significant promise for high-precision detection in biochemical and chemical sensing. However, achieving high sensitivity in low-refractive-index (RI) aqueous environments remains a formidable challenge due to weak light-matter interactions. To address this limitation, this paper designs and proposes a novel dual-channel D-shaped PCF-SPR sensor tailored for the refractive index range of 1.34–1.40. The sensor incorporates a dual-layer gold/titanium dioxide film, with gold nanoparticles deposited on the surface to synergistically enhance both propagating and localized surface plasmon resonance effects. Furthermore, a D-shaped polished structure integrated with double-sided microfluidic channels is employed to significantly strengthen the interaction between the guided-mode electric field and the analyte. Finite element method simulations demonstrate that the proposed sensor achieves an average wavelength sensitivity of 5733 nm/RIU and a peak sensitivity of 15,500 nm/RIU at a refractive index of 1.40. Notably, the introduction of gold nanoparticles contributes to an approximately 1.47-fold sensitivity enhancement over conventional structures. This work validates the efficacy of hybrid plasmonic nanostructures and optimized waveguide design in advancing RI sensing performance.

## 1. Introduction

Surface Plasmon Resonance (SPR) is a highly sensitive optical detection technique based on the collective oscillation coupling phenomenon between light and free electrons at metal-dielectric interfaces [1]. Its principle involves the excitation of surface plasmon waves (SPW) at the metal-dielectric interface under specific conditions when incident light strikes it. This excites surface plasmon waves (SPW), causing significant changes in reflected light intensity or propagation mode loss, thereby enabling high-precision detection of environmental refractive index variations. SPR sensors offer advantages such as label-free operation, rapid response, high sensitivity, and strong real-time monitoring capabilities [2]. They have been widely applied in biomolecular recognition [3], drug screening [4], environmental monitoring [5], food safety [6], and clinical diagnostics. SPR sensing provides distinct advantages over MPI and electrochemical approaches in terms of label-free operation, real-time response, and non-contact optical interrogation. In recent years, with the advancement of fiber optic technology, integrating the SPR effect into photonic crystal fibers (PCF) has become a research hotspot. PCF offers flexible geometric structures and strong light confinement capabilities. By designing the structure to regulate the coupling efficiency between the guided mode and the metal film, the performance of SPR sensors can be enhanced.

To further enhance the sensitivity of SPR sensors, researchers have incorporated nanoparticles, particularly gold nanoparticles (AuNPs), leveraging their localized surface plasmon resonance (LSPR) and the electromagnetic coupling effect with the surface plasmon of the gold film [7]. This approach amplifies the local electric field strength, thereby improving the sensor’s responsiveness to refractive index changes. In 2018, Zhang et al. [8] proposed a novel strategy to enhance SPR sensitivity by introducing a TDNP coating on the gold film surface to modify the plasmonic interface. This approach achieved a sensitivity of up to 2567 nm/RIU, representing an approximately 1.38-fold improvement over conventional unmodified gold film SPR structures. In 2020, Miliutina et al. [9] immobilized gold nanoparticles with sharp edges onto a gold film surface on a multimode fiber, significantly improving sensor performance by increasing sensitivity from 1827 nm/RIU to 3164 nm/RIU. In the same year, Niu et al. [10] designed a D-shaped PCF-SPR sensor with gold nanocapsules modified on the gold film surface, achieving an average sensitivity of 3064 nm/RIU within the refractive index range of 1.3323–1.3722. In 2021, Wang et al. [11] further enhanced SPR coupling by introducing gold nanorod structures onto the gold film surface, achieving a maximum sensor sensitivity of 4465 nm/RIU. In 2025, Li et al. [12] proposed an SPR sensor based on an Ω-shaped structure. By introducing a core–shell structure composed of gold nanoparticles (AuNPs) coated with polydimethylsiloxane (PDMS) in the sensitive region, they achieved simultaneous detection of refractive index (RI) and temperature (T), with a high sensitivity of 2760 nm/RIU. PDMS-modified Ag film, enabling simultaneous detection of refractive index (RI) and temperature (T) with a high sensitivity of 2760 nm/RIU.

Gold nanoparticles demonstrate considerable potential for enhancing SPR sensor performance, as evidenced by their efficacy in local electric field enhancement and resultant sensitivity improvement. Nevertheless, existing designs still exhibit room for optimization in structural configuration and coupling mechanisms, particularly in achieving effective synergy among fiber architecture, film composition, and nanoparticle arrangement. To address this challenge, this paper proposes a D-shaped double-side grooved PCF-SPR sensor with an integrated TiO_2_/Au bilayer film and a gold nanoparticle (AuNP) array. The symmetrical grooves on both sides of the fiber are designed to enlarge the coupling region near the metal–dielectric interface. The TiO_2_ layer acts as an adhesive and coupling-enhancing interlayer between the fiber core and the Au film, which serves as the primary plasmon excitation layer. Furthermore, an array of AuNPs is introduced on the Au film to strengthen the synergistic interaction between localized and propagating surface plasmons. Numerical simulations reveal that the structure without AuNPs achieves an average sensitivity of 4233 nm/RIU in the refractive index range of 1.34–1.40, which increases significantly to 5733 nm/RIU after AuNP modification. This study systematically examines the influence of electric field distribution, film parameters, and nanoparticle geometry on sensor performance [13,14,15]. The proposed design is also compared with several existing sensor configurations, demonstrating its promising potential for high-sensitivity refractive index detection in biochemical applications.

## 2. Structural Design and Theoretical Models

### 2.1. Physical Mechanism of Plasmonic Enhancement

Surface plasmon resonance (SPR) in the proposed sensor originates from the phase-matching condition between the core-guided modes of the photonic crystal fiber (PCF) and the surface plasmon polariton (SPP) mode supported at the metal–analyte interface. Owing to the side-polished D-shaped geometry, a portion of the guided optical field extends beyond the fiber core and penetrates into the plasmonic region, enabling efficient coupling to the SPP mode when their propagation constants become comparable.

The dual-channel D-shaped PCF configuration provides additional degrees of freedom for mode engineering. By tailoring the air-hole distribution and polishing depth, the effective refractive index and field distribution of the guided modes can be precisely controlled, thereby improving the overlap between the evanescent field and the plasmonic interface. Compared with conventional single-channel or planar SPR configurations, this structure enhances the coupling efficiency and broadens the accessible refractive index sensing range.

The introduction of a TiO_2_ interlayer between the gold film and the silica substrate plays a critical role in modulating the plasmonic response. Due to its relatively high refractive index, the TiO_2_ layer acts as an optical transition medium that facilitates stronger penetration of the evanescent field into the metal layer. This leads to improved wavevector matching between the guided modes and the SPP mode, resulting in enhanced resonance strength and tunable resonance wavelength. In addition, the TiO_2_ layer contributes to improved structural stability and adhesion of the gold film, which is beneficial for practical implementations.

Gold nanoparticles (AuNPs) deposited on the gold film further enhance the sensing performance through localized surface plasmon resonance (LSPR). When illuminated, AuNPs support highly confined localized electromagnetic fields in their vicinity, particularly within the nanoscale gaps between the nanoparticles and the underlying gold film. The near-field coupling between LSPR and the propagating SPP mode gives rise to a hybrid plasmonic resonance, significantly amplifying the local electric field intensity at the sensing interface. As a result, small variations in the analyte refractive index induce pronounced shifts in the resonance wavelength.

It should be noted that, although an idealized periodic arrangement of AuNPs is assumed in the simulations, the overall sensing behavior is primarily governed by the average nanoparticle coverage and collective plasmonic coupling rather than the exact particle positions. Therefore, the proposed enhancement mechanism is expected to remain robust against moderate randomness and slight aggregation in practical fabrication scenarios.

Overall, the synergistic integration of the dual-channel D-shaped PCF architecture, the TiO_2_/Au bilayer, and the AuNP-induced LSPR–SPP hybridization forms the physical basis for the observed sensitivity enhancement. This mechanism provides a clear design rationale for the proposed sensor and distinguishes it from conventional SPR platforms based on planar or single-layer plasmonic structures.

### 2.2. Sensor Structural Design

**Figure 1:**
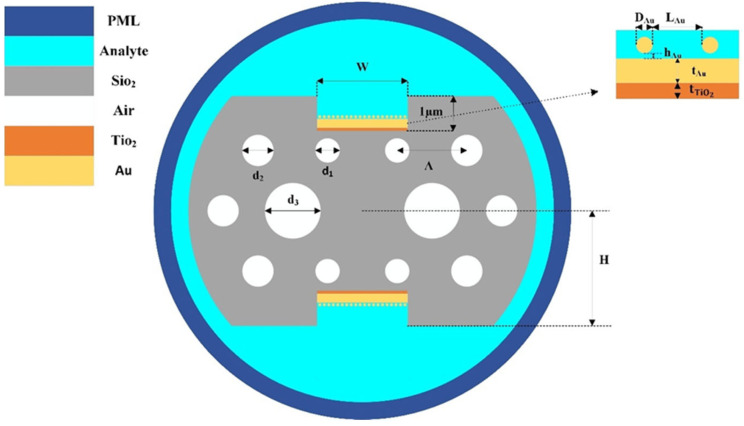
Cross-sectional view of D-shaped dual-sided slotted photonic crystal fiber surface plasmon resonance sensor.

This paper proposes a photonic crystal fiber surface plasmon resonance (PCF-SPR) sensor based on a D-shaped double-sided grooved structure. Its core structure consists of a specially designed photonic crystal fiber (PCF) and an optimized metal film composite layer, with a cross-sectional view shown in Figure 1. Quartz was selected as the substrate material for the PCF, with symmetrical polishing on both upper and lower surfaces to form two parallel D-shaped planes. The distance H between these planes and the fiber center is 2.3 µm. This D-shaped structure not only enhances the leakage effect of the core mode field but also provides an ideal platform for subsequent metal film integration. To further optimize light-plasmon coupling efficiency, square trench channels with a depth of 1 µm and width W = 2.6 µm were etched onto the D-shaped planes. This trench structure precisely constrains the deposition position of the metal film, reducing the film area while bringing the sensing region closer to the fiber core. This arrangement enhances the interaction strength between the evanescent field and surface plasmons.

The air holes within the PCF feature a double-layer hexagonal arrangement. The inner layer consists of air holes with a diameter of d_3_ = 1.5 µm positioned on both sides of the fiber core, while the upper and lower layers comprise straight air holes with a diameter of d_1_ = 0.7 µm. This asymmetric design regulates the mode field distribution. The outer air holes employ a periodic arrangement with a diameter of d_2_ = 1.3 µm and a lattice constant of Λ = 1.9 µm, serving to adjust the fiber’s effective refractive index and optimize light field confinement. A composite metal film composed of TiO_2_ and Au is sequentially deposited at the groove bottom, with the Au layer acting as the core functional layer for SPR at a thickness of t_Au_ = 40 nm. The TiO_2_ layer (thickness t_TiO2_ = 8 nm) serves as an adhesive layer, not only enhancing the adhesion between the Au film and the fiber substrate but also improving the excitation efficiency of surface plasmons due to its high refractive index. The thickness is set to t_Au_ = 40 nm. Additionally, the Au film surface is modified with gold nanoparticles (AuNPs), which further enhance the sensing signal by utilizing their localized surface plasmon resonance effect.

### 2.3. Material Refractive Index Model

The substrate material selected for this study is SiO_2_, whose refractive index in the visible to near-infrared wavelength range can be calculated using the Sellmeier equation [16]:

(1) $$n^{2}\left(\lambda \right)=1+\frac{0.696\;\lambda^{2}}{\lambda^{2}-0.0047}+\frac{0.408\;\lambda^{2}}{\lambda^{2}-0.014}+\frac{0.897\;\lambda^{2}}{\lambda^{2}-97.934}$$

where *n* denotes the refractive index RI of SiO_2_, which varies with the wavelength *λ* of the incident light. In the sensor layer design, gold (Au) is selected as the excitation material to achieve surface plasmon wave (SPW) excitation, serving as one of the core materials determining SPR performance. The dielectric constant of gold can be obtained via the Drude-Lorentz model [17]:

(2) $$\varepsilon_{\mathrm{Au}}=\varepsilon_{\infty }-\frac{\omega_{D}^{2}}{\omega \left(\omega +j\gamma_{D}\right)}-\frac{\Delta \varepsilon \;\Omega_{L}}{\left(\omega^{2}-\Omega_{L}^{2}\right)+j\Gamma_{L}\omega }$$

Among these, *ε*_Au_ is the dielectric constant of gold; *ε*_∞_ is the high-frequency dielectric constant of gold; *ω* is the angular frequency of the incident light; *ω*_D_ and *γ*_D_ are the plasma frequency and damping frequency, respectively; ∆*ε* is the weighting factor; *Ω*_L_ and *Γ*_L_ correspond to the Lorentz oscillator strength and spectral width, respectively. Specific reference values are: *ε*_∞_ = 5.9673, *γ*_D_/2π = 15.92 THz, *ω*_D_/2π = 2113.6 THz, ∆*ε* = 1.09, *Γ*_L_/2π = 104.86 THz, *Ω*_L_/2π = 650.07 THz.

TiO_2_ serves as an adhesion and transition layer between the gold film and quartz, exhibiting a high refractive index. The refractive index (RI) of TiO_2_ can be calculated using the following formula [18]:

(3) $$n_{{\mathrm{TiO}}_{2}}^{2}=5.913+\frac{2.441\times 10^{7}}{\lambda^{2}-0.803\times 10^{7}}$$

### 2.4. Simulation Boundary Conditions and Modeling

**Figure 2:**
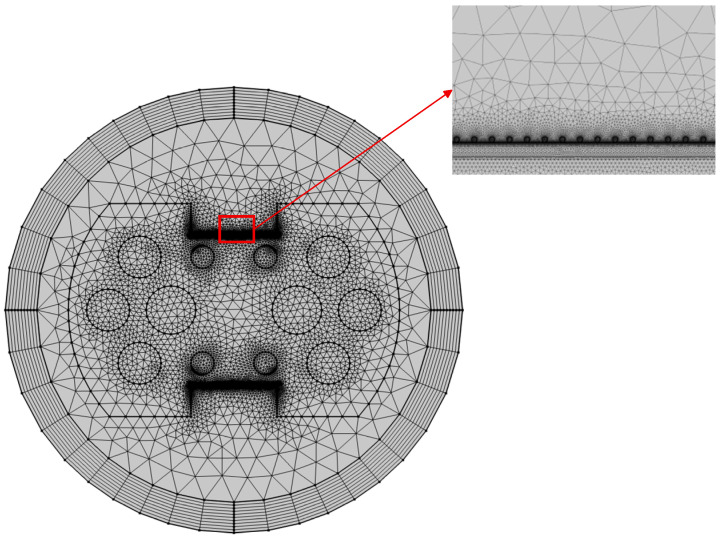
The cross-sectional mesh division of the proposed PCF-SPR sensor structure.

This study employs the Frequency Domain Wave Optics Module in COMSOL Multiphysics 6.1 to perform numerical simulations of the designed D-shaped double-sided slotted PCF-SPR sensor structure using a two-dimensional finite element method (FEM). To prevent reflected waves from interfering with energy distribution within the computational domain, a Perfectly Matched Layer (PML) with a thickness of 0.5Λ was applied around the model. This layer effectively absorbs outgoing waves while simulating open boundary conditions. A Scattering Boundary Condition was also applied to ensure normal energy emission without generating spurious reflections. To accurately resolve high-gradient electromagnetic field variations at the gold-TiO_2_ interface while maintaining overall simulation efficiency, local mesh refinement was applied to the film structure and the region surrounding the gold nanoparticles, as shown in Figure 2, thereby enhancing modeling precision in the microstructure region.

In the fabrication process, identical-sized solid rods and capillary tubes are stacked according to the hexagonal lattice arrangement in the designed structure to form PCF preforms. Following drawing, symmetrical D-shaped grooves are prepared on both sides of the fiber via mechanical polishing [19] and femtosecond laser technology [20]. Subsequently, a uniform layer of TiO_2_ is deposited over the slot region via physical vapor deposition (PVD) [21] or magnetron sputtering [22] to serve as an adhesion layer, followed by a gold (Au) film as the plasma excitation layer. Following film deposition, gold nanoparticles (AuNPs) were self-assembled onto the Au film surface [23] to enhance the localized electric field and improve sensing sensitivity. This ultimately formed a D-shaped PCF-SPR sensor featuring a dual-layer metal film with nano-enhanced structures. The simulated gold nanoparticle arrangement and parameter assumptions in this work are based on ideal conditions. In actual fabrication, AuNP colloids are typically synthesized via chemical reduction methods (e.g., trisodium citrate reduction) [24], followed by transfer onto the gold film surface through methods such as drop coating [25] or self-assembly. Since particle distribution and spacing are susceptible to factors like solvent evaporation, surface modification, and particle agglomeration [26], achieving structural uniformity in experiments is challenging to fully replicate the simulation model. Consequently, performance parameters such as sensitivity and resonance wavelength may deviate from theoretical predictions.

Gold nanoparticles (AuNPs) were prepared using the classical trisodium citrate reduction method, which is widely adopted due to its simplicity and good reproducibility. In a typical procedure, an aqueous solution of chloroauric acid (HAuCl_4_) was heated to boiling under continuous stirring, followed by the rapid addition of trisodium citrate solution. The reduction of Au^3+^ ions led to a gradual color change of the solution from pale yellow to wine red, indicating the formation of colloidal Au nanoparticles. The average nanoparticle size was mainly controlled by the molar ratio of trisodium citrate to HAuCl_4_. After synthesis, the AuNP colloidal solution was allowed to cool to room temperature and subsequently transferred onto the gold-coated sensing region of the photonic crystal fiber. Two commonly used deposition approaches were considered: drop-casting and self-assembly. For drop-casting, a small volume of the AuNP solution was carefully dropped onto the polished sensing surface and dried naturally, allowing nanoparticles to randomly distribute on the gold film. For self-assembly, the gold surface was first functionalized with an appropriate linker layer to promote electrostatic or chemical adsorption of AuNPs, leading to a quasi-uniform surface coverage. In both cases, the resulting AuNP distribution corresponds to an average surface density consistent with the simulation model. This work focuses on numerical analysis and structural optimization; references to fabrication techniques are provided only to indicate experimental feasibility.

## 3. Simulation Results and Discussion

### 3.1. Performance Analysis of PCF-SPR Without Gold Nanoparticle Modification

**Figure 3:**
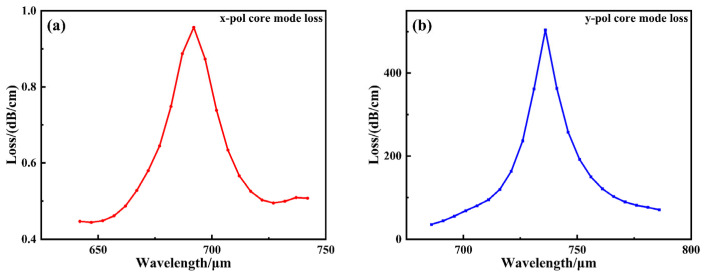
Loss spectrum of x-polarization and y-polarization when *n* = 1.385. (**a**) The loss spectrum of the core mode under x-polarization; (**b**) The loss spectrum of the core mode under y-polarization.

In the designed D-type double-slotted PCF-SPR structure, the waveguide exhibits two orthogonal polarization modes: x-polarized and y-polarized. However, as shown in Figure 3, simulation results indicate that, under the given structural parameters and incident conditions, the peak loss of the x-polarized core mode is less than 1% of that of the y-polarized mode, suggesting that the y-polarized core mode experiences low transmission loss at non-resonant wavelengths and is less susceptible to interference. The y-polarized mode exhibits stronger electric field coupling capability and energy dissipation characteristics near the metal interface, making it the dominant mode responsible for inducing the surface plasmon resonance (SPR) effect. In contrast, the x-polarized mode exhibits weaker energy coupling, and its corresponding loss variation can be neglected during the sensing process. Therefore, to highlight the dominant coupling mechanism and simplify the analysis, this paper focuses solely on the contribution of the y-polarized core mode to sensing performance, omitting detailed discussion of the x-polarized mode.

In this work, structural optimization is carried out using a single-parameter variation strategy around the SPR phase-matching condition. This approach is motivated by the fact that different structural parameters primarily influence distinct physical aspects of the sensing mechanism, such as guided-mode dispersion, evanescent field strength, and plasmonic coupling. Within the optimized operating region, the first-order interdependence among these parameters is relatively weak, allowing single-parameter perturbations to provide reliable guidance for identifying effective parameter configurations.

**Figure 4:**
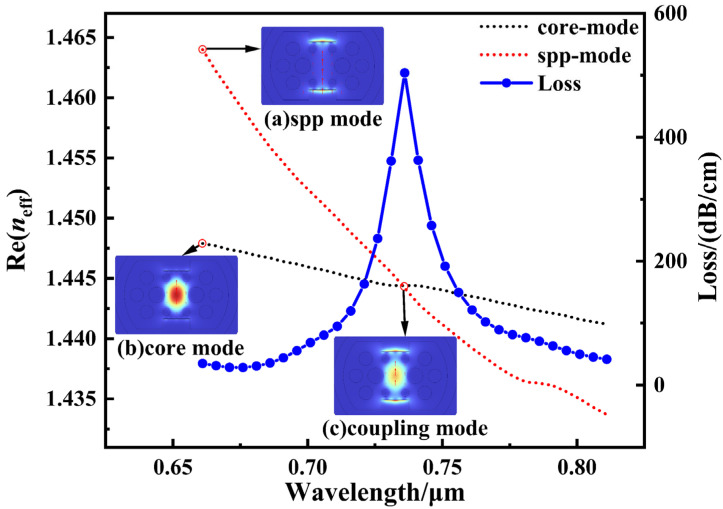
The dispersion relationship between the core mode and the SPP mode and the loss spectrum when the RI of the analyte is 1.385.

As shown in Figure 4, the figure depicts the wavelength-dependent curves of the effective refractive index real part for the SPP mode and the core mode when the analyte’s RI is 1.385, along with the corresponding mode loss spectrum. The SPR effect arises from phase-matching conditions between the two modes: when the real part of the effective refractive index of the guided mode equals that of the surface plasmon polariton (SPP) excited at a specific wavelength on the metal surface, strong coupling occurs. At this point, energy efficiently transfers from the guided mode to the SPP mode, forming surface plasmon resonance. This resonance point appears in the figure near the intersection of the guided mode and SPP mode refractive index curves, coinciding with the location of a prominent peak in the loss spectrum curve. Due to intense energy coupling and radiative loss at this wavelength, the propagation loss of the guided mode increases sharply at this point, resulting in a distinct, sharp resonance peak in the loss curve. Thus, the wavelength position of the loss peak precisely corresponds to the location of SPR occurrence. The associated refractive index change can be utilized for highly sensitive environmental refractive index detection. Figure 4a–c in the diagram correspond to the electric field distributions of the SPP mode, core mode, and phase-matched mode, respectively, with red arrows indicating the direction of the electric field.

In the following subsections, systematic simulation analyses based on the finite element method will be conducted for different structural parameters to investigate their impact on the loss spectrum characteristics. This approach aims to optimize the design and determine the optimal structural parameter configuration for the sensor.

The loss intensity depicted in the figure reflects the degree to which guide mode energy is coupled into the SPP mode and can be calculated using the following formula:

(4) αLoss=8.686×2πλ×Imneff×104

In the equation: Im(*n*_eff_) is the imaginary component of the effective RI.

### 3.2. Influence of Fiber and Film Parameters on PCF-SPR Without Gold Nanoparticle Modification

**Figure 5:**
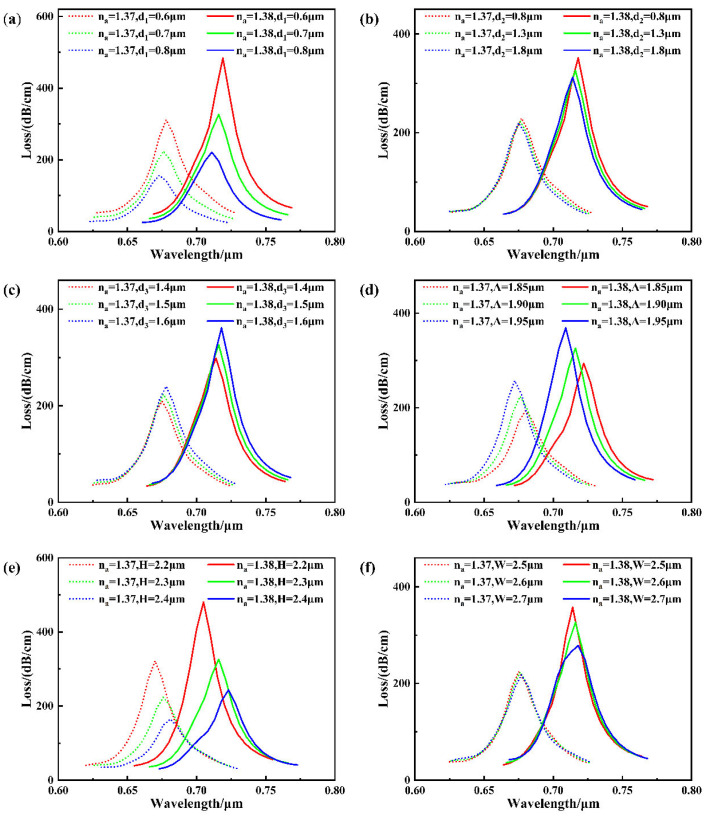
The influence of different fiber structure parameters on the loss spectrum when the RI of the analyte is 1.37 and 1.38. (**a**) d_1_; (**b**) d_2_; (**c**) d_3_; (**d**) Λ; (**e**) H; (**f**) W.

The structural parameters of photonic crystal fibers primarily include pore diameters (d_1_, d_2_, d_3_), pore spacing Λ, polishing depth H, and groove width W. These parameters mainly influence the guided mode field distribution and the contact area with the gold film. Figure 5 illustrates the effect of different photonic crystal fiber structural parameters on the core mode loss spectrum for analyte refractive indices of 1.37 and 1.38.

Figure 5a illustrates that enlarging the pore diameter $d_{1}$ adjacent to the metal layer results in a gradual reduction in the loss peak. This occurs because the reduced overlap area between the guiding mode field and the gold film weakens the coupling strength, thereby diminishing the excitation efficiency of surface plasmon resonance (SPR) and resulting in a reduced loss peak amplitude.

The resonance conditions for SPR can be described by the following wave vector matching relationship:

(5) $$k_{core}=k_{SPP}$$

where *k*_core_ and *k*_SPP_ denote the wave vectors for the core mode and surface plasmon polariton, respectively, with *k*_core_ expressed as:

(6) $$k_{core}=\frac{2\pi }{\lambda }\times n_{eff}$$

where *λ* is the wavelength of the incident light, and *n*_eff_ is the effective refractive index of the guided mode, which depends on the fiber structure. *k*_SPP_ is expressed as:

(7) $$k_{\mathrm{SPP}}=\frac{2\pi }{\lambda }\sqrt{\frac{\varepsilon_{m}\varepsilon_{d}}{\varepsilon_{m}+\varepsilon_{d}}}$$

where *ε*_m_ is the complex permittivity of the metal, and *ε*_d_ is the permittivity of the dielectric medium, such as the analyte.

This behavior arises because a larger $d_{1}\;$ causes the guided mode field to retreat further from the gold film interface, thereby reducing the effective refractive index *n*_eff_. To maintain phase-matching conditions, compensation can only be achieved by reducing the wavelength *λ*, resulting in a blue shift of the resonance wavelength. Furthermore, as shown in Figure 5a, when the analyte refractive index (RI) varies from 1.37 to 1.38, the corresponding resonance wavelength shifts by 41 nm, 40 nm, and 38 nm for d_1_, respectively. Considering key performance metrics such as loss intensity, resonance wavelength, and sensitivity, selecting d_1_ = 0.7 μm achieves an optimal balance between sensitivity and coupling efficiency. Therefore, this value is adopted as the optimized design parameter in this study.

The influence of the outer pore diameter $d_{2}$ on the loss characteristics is presented in Figure 5b, where noticeable variations in the loss spectrum are observed as $d_{2}$ increases. The loss peak gradually decreases, and the resonance wavelength continuously shifts toward the blue end of the spectrum. Further observation reveals that when the analyte refractive index increases from 1.37 to 1.38, the wavelength shifts are 41 nm, 40 nm, and 39 nm, respectively, indicating a slight decrease in structural sensitivity with increasing d_2_. This change is primarily attributed to the outer layer pores’ regulatory effect on the overall cladding environment of the fiber. As d_2_ increases, the air content in the fiber cladding rises, lowering the effective refractive index of the overall cladding. This enhances confinement of the guided mode, concentrating its energy more within the core region and away from the metal film interface. Selecting d_2_ = 1.3 μm maintains high mode field confinement and stable loss intensity while avoiding significant sensitivity degradation.

As depicted in Figure 5c, increasing the diameter $d_{3}$ of the inner left and right end pores markedly alters the loss response of the sensor. Specifically, the loss peak becomes progressively stronger, accompanied by a continuous red shift of the resonance wavelength. This change is primarily attributed to the regulatory effect of d_3_ on the horizontal expansion characteristics of the guided mode. As the pore diameters on both sides increase, the transverse symmetry of the fiber core enhances, causing the light field to spread more horizontally. Due to structural constraints, the energy from this lateral expansion is “squeezed” into the vertical direction, thereby strengthening the electric field component of the guided mode near the metal film region. This can be interpreted as the enlarged d_3_, through the spatial compression of the mode field by lateral pores, facilitating easier vertical coupling of the light field to the gold film surface. This enhances the coupling efficiency between the guided mode and the gold film, intensifying SPR excitation strength, which manifests as increased loss and a red shift in the resonance wavelength. When the analyte refractive index increases from 1.37 to 1.38, the wavelength shifts are 39 nm, 40 nm, and 40 nm, respectively. Considering both sensitivity and peak loss, d_3_ =1.5 μm is selected as the design parameter for this iteration.

The effect of the lattice constant Λ of the inner pore layer on the loss spectrum is shown in Figure 5d. When Λ is increased from 1.4 μm to 1.6 μm, the loss peak gradually intensifies, while the resonance wavelength exhibits a pronounced blue shift. Simultaneously, under conditions where the analyte refractive index is 1.37 and 1.38, the resonance wavelength shifts by 42 nm, 40 nm, and 37 nm, respectively, indicating a gradual decrease in sensitivity as Λ increases. This phenomenon can primarily be attributed to the regulation of guided mode confinement and propagation paths by the pore spacing. As Λ increases, the distance between the core and cladding expands, causing the light field distribution to spread outward. This facilitates energy leakage toward the metal film, enhancing coupling with the gold layer and intensifying SPR excitation, manifested as increased loss. Concurrently, the effective refractive index *n*_eff_ of the guided mode decreases. According to the wave vector matching equation, the system requires shorter wavelengths to achieve matching with k_SPP_, thereby inducing a blue shift in the resonance wavelength. Furthermore, as the light field expands, the electric field distribution within the sensing region becomes more dispersed. This weakens the modulation effect of refractive index changes on the resonance wavelength, consequently reducing sensitivity. Considering the balance between loss intensity, resonance wavelength stability, and sensitivity performance, selecting Λ = 1.5 μm achieves an optimal equilibrium between SPR excitation efficiency and sensing capability. Therefore, this value is adopted as the optimized design parameter for subsequent analysis in this study.

Figure 5e demonstrates that increasing the polishing depth H enlarges the vertical separation between the fiber core and the gold film, which directly influences the coupling strength between the guided mode and the surface plasmon mode. This reduces the field overlap area between the guided mode and the gold film, thereby weakening the coupling strength and decreasing the SPR excitation efficiency, manifested as a reduced loss peak. Simultaneously, the mode field distribution becomes more concentrated within the fiber core region, increasing the effective refractive index *n*_eff_ of the guided mode. According to the wave vector matching relationship, to maintain matching with the SPP wave vector, the system requires a longer wavelength to excite SPR, resulting in a resonance wavelength red shift. Furthermore, during the increase in analyte refractive index from 1.37 to 1.38, the resonance wavelength shifts were 35 nm, 40 nm, and 42 nm, respectively, indicating enhanced sensitivity with increasing H content. Considering both loss peak and sensitivity, 2.3 μm is selected as the optimal parameter value. Tolerance variation of groove depth within ±1 µm results in wavelength shift < 10 nm and sensitivity change < 500 nm/RIU; variation within ±10 nm results in changes still within acceptable range for accurate sensing.

The slot width (W) regulates the effective coupling area between the metal film and the adhesive layer, as well as the distribution path of the guided mode. Figure 5f illustrates the effect of slot width on the loss spectrum. It can be observed that the resonance wavelength redshifts as W increases. This occurs because a larger slot width expands the sensing area, facilitating easier coupling between the two modes. However, as W increases, the lateral extension area of the gold film widens, increasing the proportion of the gold film region distant from the fiber core while relatively reducing the coupling area near the core. This weakens the effective coupling strength between the guided mode and the gold film, thereby decreasing SPR excitation efficiency and lowering the loss peak. When W increases from 2.5 μm to 2.7 μm, the resonance wavelength shifts by 39 nm, 40 nm, and 41 nm for an analyte RI change from 1.37 to 1.38. Balancing coupling strength and sensitivity, selecting W = 2.6 μm achieves an optimal equilibrium between loss intensity and sensing performance.

**Figure 6:**
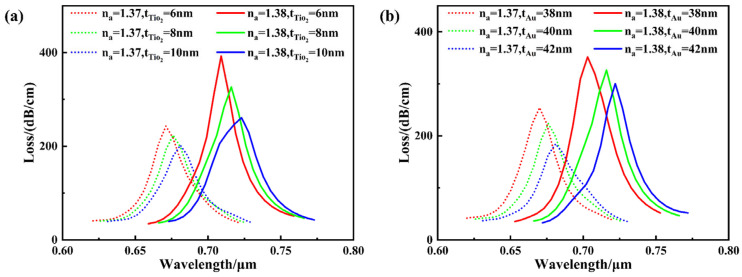
When the refractive index (RI) of the analyte is 1.37 and 1.38, the effects of different fibe r structure parameters on the loss spectrum. (**a**) t_TiO2_; (**b**) t_Au_.

The membrane structure employed in this design consists of a composite film formed by a gold layer and a TiO_2_ layer, with key parameters being the gold layer thickness t_Au_ and the TiO_2_ layer thickness t_TiO2_. Figure 6 illustrates the impact of variations in these membrane parameters on the loss spectrum, considering analyte refractive indices of 1.37 and 1.38.

A thin TiO_2_ layer is introduced between the Au film and the PCF surface, serving as an adhesion and dielectric buffer layer. Owing to its good chemical compatibility with silica and improved interfacial bonding with gold, the TiO_2_ layer significantly enhances the mechanical stability of the Au film. Moreover, the high refractive index of TiO_2_ facilitates better phase matching between the guided core mode and the surface plasmon polariton mode, thereby contributing to improved SPR excitation and sensing performance. Figure 6a illustrates the effect of TiO_2_ film thickness variation on the loss spectrum. As the TiO_2_ film thickness increases from 6 nm to 10 nm, the sensor’s resonance wavelength gradually redshifts, while the loss peak continuously decreases. while the resonance wavelength shift at refractive indices of 1.37 and 1.38 increased from 38 nm to 42 nm, indicating enhanced sensitivity with increased film thickness. This phenomenon arises because thicker films increase the equivalent refractive index at the metal-dielectric interface, thereby increasing the surface plasmon polariton (SPP) wave vector k_SPP_. To satisfy the wave vector matching condition, the system requires excitation at longer wavelengths, leading to wavelength red-shift. Conversely, thicker films reduce the coupling efficiency between the guided electric field and the gold film, weakening SPR excitation intensity and manifesting as a decrease in the loss peak. A TiO_2_ film thickness of 8 nm achieves an optimal balance between sensitivity and coupling efficiency, making it the selected parameter for this design.

Gold serves as the metal layer in SPR that excites surface plasmon resonance, playing a crucial role in providing free electrons and supporting the propagation of surface plasmon waves. Figure 6b illustrates the impact of gold film thickness on its sensing performance. When the Au film thickness increased from 38 nm to 42 nm, the sensor’s resonance wavelength gradually shifted toward the red end of the spectrum, and the loss peak gradually decreased. Under conditions where the analyte refractive index increased from 1.37 to 1.38, the resonance wavelength shifts were 33 nm, 40 nm, and 41 nm, indicating that sensitivity increases with film thickness before reaching saturation. This phenomenon arises because thickening the gold film enhances the equivalent refractive index at the metal/dielectric interface. To satisfy the wave vector matching condition, the system requires a longer wavelength, leading to resonance red-shift. Simultaneously, the thicker film suppresses the coupling of guided modes to the metal interface, resulting in a reduced loss peak. Considering both SPR excitation efficiency and wavelength sensitivity performance, a 40 nm gold film thickness achieves the optimal balance of performance characteristics and is therefore selected as the optimal parameter in this design.

### 3.3. Evaluation of Sensing Performance of PCF-SPR Without Gold Nanoparticle Modification

Sensitivity (SW) is the shift in resonant wavelength caused by a unit change in refractive index. It reflects the sensor’s responsiveness to environmental refractive index variations and is one of the most critical metrics. It can be expressed by the following formula:

(8) $$S_{W}=\frac{∆\lambda_{Peak}}{∆n}$$

**Figure 7:**
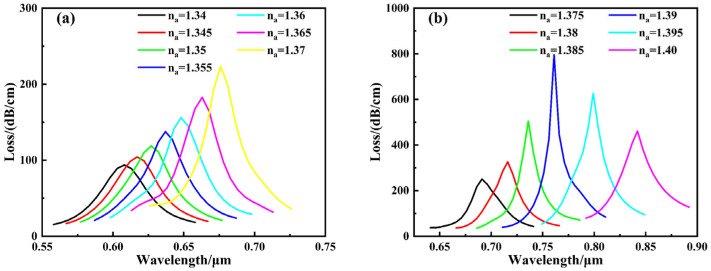
Loss curve of RI for different analytes without modification by gold nanoparticles. (**a**) 1.34–1.37; (**b**) 1.375–1.40.

The evolution of the loss spectrum with analyte refractive indices ranging from 1.34 to 1.40 is presented in Figure 7. The figure reveals that within the studied refractive index range, the resonance wavelength continuously red-shifts with increasing analyte refractive index, while the loss peak also exhibits an upward trend. When the refractive index increases to 1.39, optimal coupling is achieved between the guided mode and surface plasmon polaritons (SPPs), resulting in maximum resonance loss. Further increasing the refractive index disrupts this coupling condition, leading to reduced energy transfer efficiency and a subsequent weakening of the loss peak.

The refractive index range of 1.34–1.40 is chosen to target typical biochemical and biosensing applications involving aqueous solutions and surface-bound biomolecular interactions, while the sensing mechanism can be readily extended to other RI ranges through structural re-optimization. Numerical results indicate that when the analyte refractive index exceeds 1.40, the SPR resonance exhibits pronounced spectral broadening, leading to a reduced quality factor and decreased wavelength interrogation accuracy. This behavior can be attributed to phase-matching mismatch and increased radiative damping under high-index conditions. Therefore, the sensor structure is intentionally optimized for the refractive index range of 1.34–1.40, where a well-defined resonance dip and high sensing sensitivity are simultaneously achieved.

**Figure 8:**
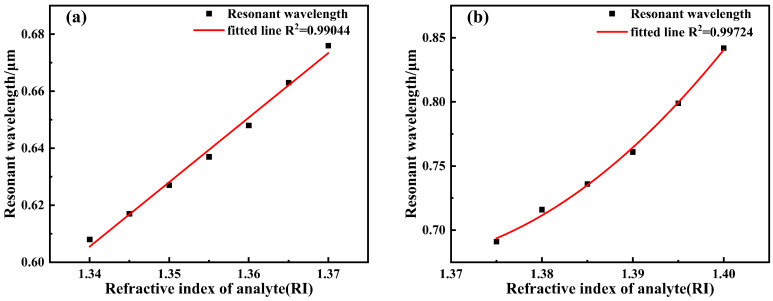
The fitting curve of the analyte RI variation. (**a**) 1.34~1.37; (**b**) 1.375~1.40.

When the analyte refractive index reaches 1.40, calculated according to Equation (8), the wavelength sensitivity at this point can reach 8600 nm/RIU, representing the maximum value across the entire refractive index detection range. Concurrently, considering the spectrometer resolution (assumed to be 0.1 nm), the corresponding detection limit is determined to be 1.16 × 10^−5^ RIU. Furthermore, the corresponding Figure of Merit (FOM) is calculated to be 215 RIU^−1^. To analyze the sensor’s response characteristics across different refractive index ranges, the analyte refractive index interval is divided into two segments for fitting, as shown in Figure 8. For the 1.34–1.37 range, the resonance wavelength exhibits a good linear relationship with refractive index, achieving a linear correlation coefficient of 0.99044 and an average sensitivity of 2267 nm/RIU. In the higher refractive index segment (1.375–1.40), the relationship between resonance wavelength and refractive index became nonlinear. At higher refractive indices (RI > 1.375), the SPR dispersion deviates from linearity due to phase-matching nonlinearity and plasmonic mode redistribution at the metal–analyte interface. As a result, the resonance wavelength exhibits a nonlinear dependence on the analyte refractive index. Although nonlinear behavior appears in the high refractive index region, it remains highly predictable and can be effectively addressed through polynomial calibration, which is commonly adopted in high-sensitivity SPR sensing systems. After fitting with a second-order polynomial, the correlation coefficient reached 0.99724, demonstrating excellent fitting performance, with an average sensitivity of 6040 nm/RIU. Overall, across the entire 1.34–1.40 range, the sensor’s average sensitivity was 3900 nm/RIU.

### 3.4. Performance Analysis of PCF-SPR Modified with Gold Nanoparticles

**Figure 9:**
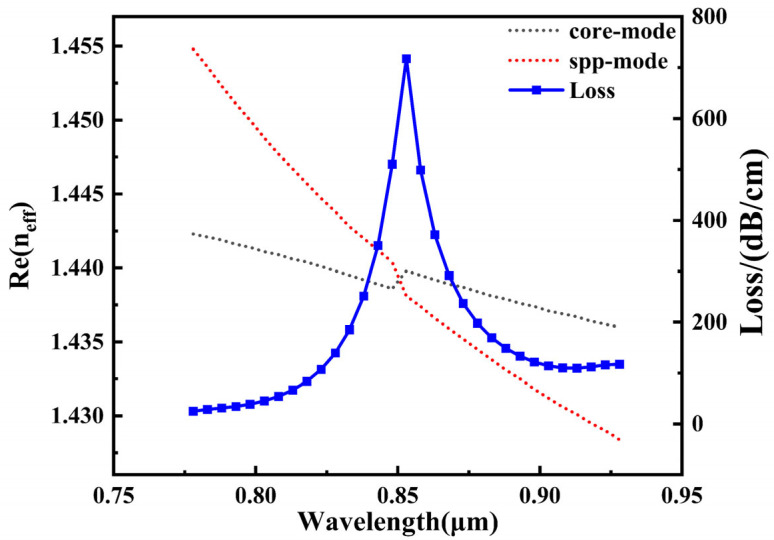
When the analyte RI = 1.385, the dispersion relationship between the core mode and the SPP mode and the loss spectrum.

Figure 9 correlates the wavelength-dependent real part of the effective refractive indices of the core-guided mode and the surface plasmon polariton (SPP) mode at an analyte refractive index of 1.395, together with the associated mode loss spectrum. The figure reveals that at specific wavelengths, the effective refractive indices of the guided mode and SPP intersect, indicating phase matching and strong coupling between the two modes at these wavelengths. This results in significant energy coupling from the guided mode to the metal surface, forming a distinct surface plasmon resonance peak. Compared to the unmodified structure, this loss peak is sharper and exhibits a higher peak intensity, indicating that the introduction of gold nanoparticles effectively enhances coupling strength and energy dissipation. Furthermore, by comparing the dispersion relationship between the core mode, SPP mode, and loss spectrum in Figure 4 when the analyte RI = 1.385, it can be observed that under identical analyte refractive index conditions, the resonance wavelength of the structure modified with gold nanoparticles exhibits a pronounced red shift compared to the unmodified structure. This is primarily due to the following mechanism: when Au nanoparticles approach the gold film surface, incident light excites dipole oscillations within the particles, triggering localized surface plasmon resonance (LSPR). Simultaneously, a mirror dipole of opposite direction and intensity-related properties is induced in the gold film. This mirror dipole interacts strongly with the original dipole within the nanoparticle, forming a coupled system. Due to their opposite polarities, this interaction suppresses the recovery ability of plasmonic electrons, reducing the resonance frequency of the oscillating system [27]. This causes the resonance conditions to shift toward longer wavelengths, manifesting as a red shift in the resonance wavelength. Alternatively, it can be interpreted that gold nanoparticles are positioned within the dielectric region above the gold film. The dielectric constant of AuNPs is higher than that of the background analyte medium, effectively raising the overall dielectric constant of the system. This leads to an increase in the square root of $\sqrt{\frac{\varepsilon_{m}\varepsilon_{d}}{\varepsilon_{m}+\varepsilon_{d}}}$ within the SPP wave vector. Combined with Equation (7), matching kSPP with kcore necessitates introducing longer wavelengths, resulting in a resonance wavelength redshift. This phenomenon further validates the crucial role of gold nanoparticles in regulating the localized electromagnetic environment and enhancing SPR response.

**Figure 10:**
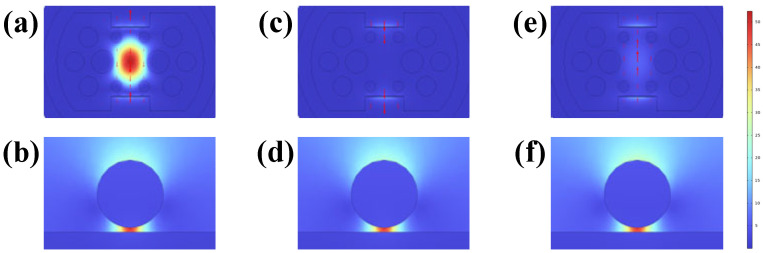
When the analyte RI is 1.385, the electric field distribution under different modes and their enlarged images of gold nanoparticles are shown. (**a**,**b**) Core mode at a wavelength of 778 nm; (**c**,**d**) SPP mode at a wavelength of 778 nm; (**e**,**f**) Coupling mode at a wavelength of 853 nm.

**Figure 11:**
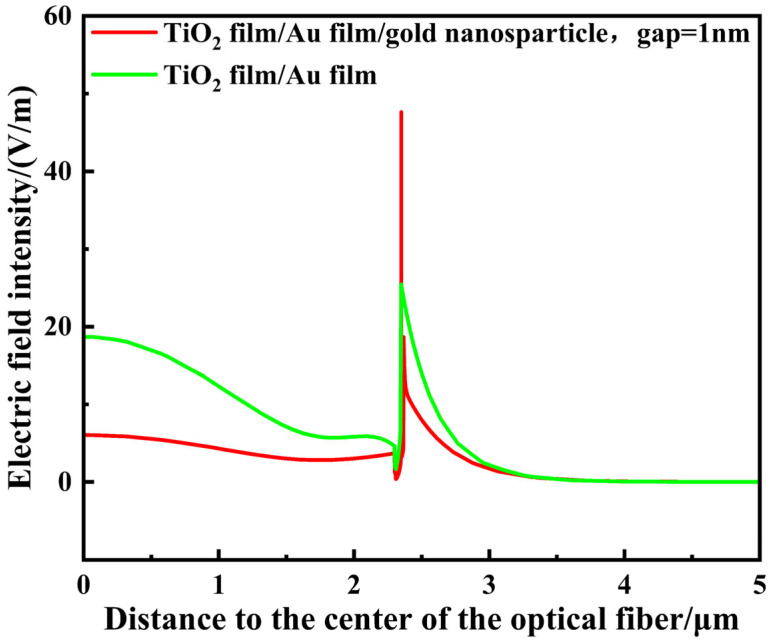
When the analyte RI = 1.385 and surface plasmon resonance occurs simultaneously, the local electric field curve of TiO_2_ film/gold film and the local electric field curve of gold nanoparticles at a gap of 1 nm.

A magnified view of the nanoscale gap between the gold film and the surface-decorated gold nanoparticles is provided in Figure 10, highlighting the spatial distribution of the electric field within this confined region. The figure reveals distinct “hot spot” regions forming between the gold nanoparticles and the gold film, where the local electric field is significantly enhanced within these nanogaps. The maximum electric field intensity exhibits a marked increase compared to the unmodified structure. This enhancement originates from the hybridization between the localized surface plasmon resonance (LSPR) excited by the gold nanoparticles and the surface plasmon polariton (SPP) modes of the gold film [28]. When the LSPR frequency approaches the SPP coupling frequency, mutual excitation forms a strongly coupled resonant mode. Constrained by the gap, the local electric field intensity at the metal–dielectric interface is significantly amplified. This enhances the interaction between light and the ambient refractive index, thereby improving the sensor’s responsiveness to minute refractive index changes. Figure 11 provides a more direct comparison of the localized electric fields generated by the TiO_2_ film/gold film/gold nanoparticle composite structure versus the TiO_2_ film/gold film structure. This demonstrates that the interaction between gold nanoparticles and the gold film plays a crucial role in enhancing the localized electric field strength, and the improvement in sensitivity primarily depends on the electric field intensity at the gold nanoparticle surface [29].

### 3.5. Influence of Parameters on Gold Nanoparticles

Gold nanoparticles demonstrate promising potential for enhancing SPR sensor performance, with this enhancement likely dependent on structural parameters such as particle diameter (D_Au_), interparticle spacing (L_Au_), and the vertical gap between particles and the gold film (h_Au_). To gain deeper insight into the role of these parameters in the enhancement mechanism, a systematic investigation of their effects on resonance characteristics and sensing performance is warranted.

**Figure 12:**
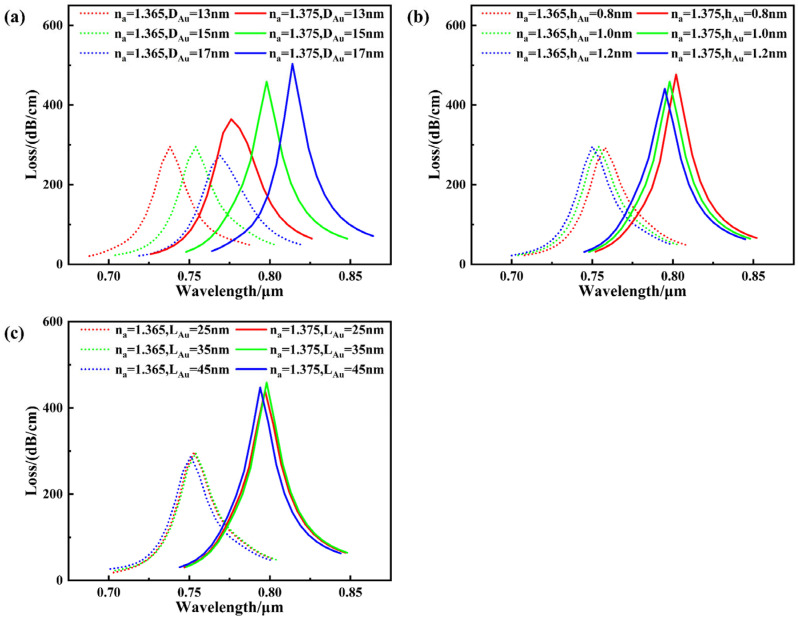
The influence of nanoparticle parameters on the loss spectrum when the analyte RI is 1.365 and 1.375. (**a**) D_Au_; (**b**) h_Au_; (**c**) L_Au_.

An increase in the gold nanoparticle diameter (D_Au_) leads to a continuous red shift of the resonance wavelength, as summarized in Figure 12a. This phenomenon arises because the oscillation frequency of the LSPR is influenced by its size; altering the nanoparticle size modulates the position of the LSPR resonance peak [30]. As the nanoparticle diameter increases, the oscillation path of free electrons within the particle lengthens, leading to a decrease in the LSPR frequency generated by the gold nanoparticles and resulting in a red shift.

At the same time, the loss peak exhibits a non-monotonic dependence on the nanoparticle size across different analyte refractive indices, as also evidenced in Figure 12a. This phenomenon may be attributed to changes in the frequency matching between LSPR and SPP modes within varying background media, leading to enhanced or diminished energy coupling efficiency and consequently affecting the intensity of the loss peak. Simultaneously, alterations in electric field distribution caused by size variations may also enhance or diminish the loss peak. Since the principles governing how interparticle spacing and the perpendicular gap between particles and the film modulate the local electric field distribution and coupling strength are analogous to size changes, relevant simulations indicate that their effects on the loss peak exhibit similar trends. Consequently, the subsequent discussion will not elaborate further on the variations in loss peak intensity induced by parameter changes. This phenomenon indicates that during structural optimization design, the influence of AuNP parameters on the loss peak must fully account for coupling behavior within the target refractive index environment, rather than relying solely on any fixed trend. For identical D_Au_ dimensions, corresponding wavelength shifts of 38, 44, and 45 nm were observed for different analyte RIs. It can be observed that as D_Au_ increases, the sensor sensitivity shows an upward trend. However, when D_Au_ increases from 15 nm to 17 nm, the improvement in sensitivity becomes less pronounced. To avoid the potential side effect of excessive resonance peak broadening caused by larger particle sizes, 15 nm was selected as the design size for the gold nanoparticles after comprehensive consideration.

With increasing vertical separation (h_Au_) between the gold nanoparticles and the metal film, the resonance wavelength progressively shifts toward shorter wavelengths, as illustrated in Figure 12b. This shift is primarily attributed to the weakening coupling between the dipole in the gold nanoparticle and the mirror dipole in the gold film as the gap increases, leading to reduced LSPR excitation efficiency. To maintain the coupling between LSPR and SPP, higher-frequency incident light must be introduced to compensate for the decreased coupling efficiency, resulting in a red shift of the resonance wavelength. The vertical distance between gold nanoparticles and the gold film inversely correlates with electric field enhancement intensity. However, sensing sensitivity does not strictly follow the simplistic rule of “higher electric field strength equating to higher sensitivity” [31]. The resonance wavelength shifts by 44, 44, and 45 nm when the refractive index changes from 1.365 to 1.375. Considering both sensitivity and controllability in practical fabrication, a gap of 1.0 nm was ultimately selected as the distance between the gold film and gold nanoparticles. It should be noted that the proposed nanoparticle-enhanced structure exhibits good tolerance to small variations in the nanoparticle–film gap, which is beneficial for practical fabrication and experimental implementation.

Figure 12c reveals a transition in the resonance behavior as the interparticle spacing (L_Au_) increases, characterized by an initial red shift followed by a subsequent blue shift of the resonance wavelength. In this system, not only does coupling occur between the dipole in the gold nanoparticle and the mirror dipole in the gold film, but coupling also occurs between the gold nanoparticles themselves [32]. When the spacing is small, the dipole coupling between gold nanoparticles is relatively strong, thereby lowering the resonance frequency of the system and shifting the resonance wavelength toward longer wavelengths. As the spacing increases, the dipole coupling rapidly weakens. This prevents the LSPR of each nanoparticle from hybridizing with the LSPR of adjacent nanoparticles, effectively isolating the LSPR of a single gold nanoparticle to interact with the SPR of the gold film. Consequently, the resonance frequency of the system increases, and the resonance wavelength shifts toward the blue. When the L_Au_ increased from 25 nm to 45 nm, the resonance wavelength shifts at refractive indices ranging from 1.375 to 1.365 were 44 nm, 44 nm, and 43 nm, respectively. This indicates that the sensor’s local sensitivity reaches its optimum at a spacing of 35 nm. Considering resonance intensity, sensitivity performance, and practical controllability of structural arrangement, 35 nm was ultimately selected as the design parameter for the gold nanoparticle spacing in this study. Among the AuNP parameters, the nanoparticle diameter plays a dominant role in determining the sensing performance, while moderate variations in interparticle spacing around the optimized value introduce only minor sensitivity fluctuations. Therefore, the tolerance analysis mainly focuses on D_Au_. Tolerance variation in AuNP size (±2 nm size) introduces wavelength shift within ±20 nm while maintaining sensitivity within ±500 nm/RIU. Tolerance analyses indicate that variations in groove depth (±1 μm) and nanoparticle size/spacing (±2 nm size, ±10 nm spacing) lead to minor resonance shifts and small sensitivity variations, demonstrating that the proposed design is robust against typical fabrication deviations.

### 3.6. Evaluation of Sensing Performance of PCF-SPR Modified with Gold Nanoparticles

**Figure 13:**
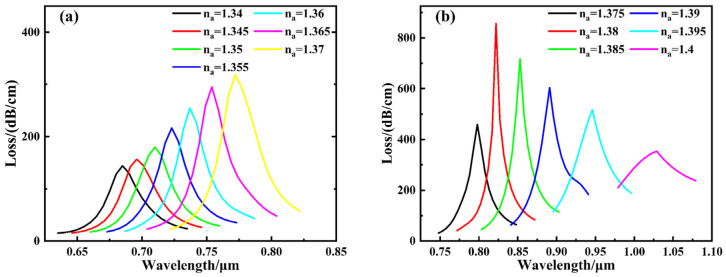
Loss curve of RI for different analytes with modification by gold nanoparticles. (**a**) 1.34–1.37; (**b**) 1.375–1.40.

**Figure 14:**
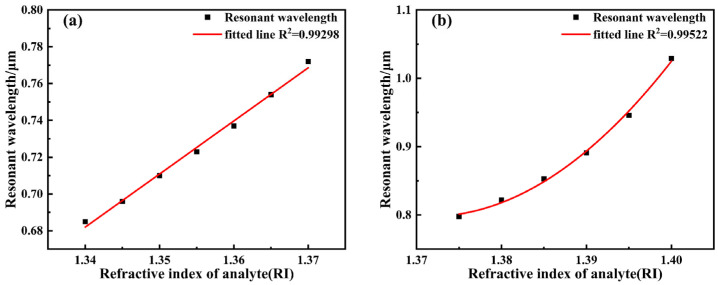
Fitting curve of analyte RI variation. (**a**) 1.34–1.37; (**b**) 1.375–1.40.

Figure 13 illustrates the effect of varying the refractive index between 1.34 and 1.40 on the loss spectrum curve. The figure reveals that within the studied refractive index range, the resonance wavelength continuously red-shifts as the analyte’s refractive index increases, while the loss peak also exhibits an upward trend. When the refractive index increases to 1.38, optimal coupling is achieved between the guided mode and surface plasmon polaritons (SPPs), resulting in maximum resonance loss. Further increasing the refractive index disrupts this coupling condition, reducing energy transfer efficiency and consequently weakening the loss peak. When the analyte refractive index reaches 1.40, the wavelength sensitivity calculated using Equation (8) achieves 16,600 nm/RIU, representing the maximum value across the entire refractive index detection range. Combined with the spectrometer resolution (assumed to be 0.1 nm), the corresponding detection limit is 6.02 × 10^−6^ RIU. Furthermore, the corresponding FOM was calculated to be 71.9 RIU^−1^. To analyze the sensor’s response characteristics across different refractive index ranges, the analyte refractive index interval was divided into two segments for fitting, as shown in Figure 14. For the 1.34–1.37 range, the resonance wavelength exhibits a good linear relationship with refractive index, achieving a linear fitting correlation coefficient of 0.99415 and an average sensitivity of 2900 nm/RIU. In the higher refractive index segment (1.375–1.40), the relationship between resonance wavelength and refractive index became nonlinear. After fitting with a second-order polynomial, the correlation coefficient reached 0.99522, demonstrating excellent fitting performance, with the average sensitivity significantly increasing to 9240 nm/RIU. Overall, across the entire 1.34–1.40 range, the sensor exhibits an average sensitivity of 5733 nm/RIU. Au nanoparticles provide an additional sensitivity enhancement on top of the optimized dual-channel D-shaped structure, resulting in a 1.47-fold improvement.

Although an ideal periodic arrangement of AuNPs is assumed in the simulations, the sensing performance is mainly governed by the average nanoparticle coverage and the hybrid plasmonic coupling between localized surface plasmons and propagating surface plasmon polaritons. Therefore, the proposed sensor response is expected to be robust against random nanoparticle distribution and mild aggregation commonly encountered in practical fabrication.

It should be noted that the finite element simulations conducted in this work are based on idealized structural assumptions, in which parameters such as the nanoparticle size, spacing, and the gap between the AuNPs and the gold film are considered to be strictly uniform and precisely controllable. However, in practical fabrication processes, these parameters are inevitably influenced by experimental methods and process precision, leading to inherent randomness and unavoidable deviations. For example, commonly used AuNP synthesis and deposition techniques, including trisodium citrate reduction, seed-mediated growth, and sol–gel-assisted deposition, may result in particle size dispersion, local aggregation, and random spatial distribution. In addition, controlling the vertical gap between nanoparticles and the gold film typically relies on molecular modification layers or template-assisted techniques, which are constrained by process stability. Consequently, fluctuations in these structural parameters may lead to quantitative discrepancies between experimental measurements and simulation predictions, while the overall sensing trends and enhancement mechanisms remain consistent after appropriate calibration.

### 3.7. Comparison with Other Enhancement Strategies

To position the proposed sensor within the context of existing enhancement strategies, Table 1 summarizes representative high-sensitivity PCF-SPR sensors reported in recent literature.

As summarized in Table 1, various enhancement strategies have been reported to improve the sensitivity of PCF-SPR sensors, including 2D material functionalization, nanostructure integration, and multilayer dielectric–metal coatings. It should be noted that direct comparison is affected by differences in refractive index range, structural configuration, and optimization targets.

## 4. Conclusions

This paper proposes a surface plasmon resonance (SPR) sensor based on a double D-shaped photonic crystal fiber enhanced with gold nanoparticles. By leveraging the hybrid resonance effects between the metal film and the nanoparticles, the sensor achieves substantial performance improvement. A systematic parameter sweep was conducted to investigate the influence of structural parameters—including air hole diameters (d_1_, d_2_, d_3_) and pitch Λ, polishing depth H, groove width W, metal film thickness, TiO_2_ interlayer thickness, and Au nanoparticle size and spacing—on SPR excitation and sensing sensitivity. Theoretical analysis based on dipole coupling, localized field enhancement, and wavevector matching mechanisms provided further insight into resonance behavior and supported structural optimization. In the present study, the proposed design focuses on refractive index sensing under isothermal or temperature-controlled conditions, where temperature-induced refractive index variations of the structural materials are negligible and analyte-related temperature effects can be addressed through calibration or compensation if required. The optimized sensor exhibits excellent performance in the refractive index range of 1.37–1.40, with a maximum sensitivity of 16,600 nm/RIU, a detection limit of 6.02 × 10^−6^ RIU, and a quality factor of 71.9 RIU^−1^, alongside favorable linearity and resonance intensity. The incorporation of gold nanoparticles induces a strong localized electric field between the particles and the gold film, significantly improving SPR excitation efficiency. This enhancement manifests as a redshift in resonance wavelength, an increase in the loss peak, and a 1.47-fold sensitivity improvement over the unmodified structure. Although the current structural parameters have been locally optimized, interdependent coupling effects remain. Global optimization will be pursued in future work to further advance sensor performance.

## Figures and Tables

**Table 1:** Comparison Table of Several Recent Enhanced PCF-SPR Sensors.

Enhancement Strategy	Structural Features	RI Sensing Range	Wavelength Sensitivity (nm/RIU)	Figure of Merit (FOM)/Resolution	Reference
Gold nanoparticles-modified PCF	TiO_2_/Au bilayer + AuNPs on double D-shaped PCF	1.34–1.40	~5733	FOM ~71.9 RIU^−1^	This work
Au–TiO_2_ bilayer PCF-SPR	D-type dual-mode PCF with Au–TiO_2_ coating; side-polished groove	1.25–1.36	5666.7	Resolution ~10^−6^ RIU	Ref. [33]
Gold nanorods-modified PCF	Au nanorods integrated with metal film	~1.33–1.37	~9000	Resolution ~10^−6^ RIU	Ref. [11]
Monolayer MoS_2_-coated PCF	Single-layer MoS_2_ on Au film	~1.33–1.40	~4500	FOM ~20–30 RIU^−1^	Ref. [17]
Graphene-coated PCF	Graphene monolayer on Au film	~1.331–1.339	~2000	FOM ~2000 RIU^−1^	Ref. [34]
Graphene–Au hybrid PCF	Graphene combined with Au film	~1.32–1.41	~4200	Resolution ~10^−5^ RIU	Ref. [35]

## Data Availability

The original contributions presented in this study are included in the article. Further inquiries can be directed to the corresponding author(s).
